# Diagnostic value of serum STIP1 in HCC and AFP-negative HCC

**DOI:** 10.1093/labmed/lmae033

**Published:** 2024-05-23

**Authors:** Haiqing Sun, Ning Liu, Jinli Lou

**Affiliations:** Department of Clinical Laboratory Center, Beijing Youan Hospital, Capital Medical University, Beijing, China; Department of Clinical Laboratory Center, Beijing Youan Hospital, Capital Medical University, Beijing, China; Department of Clinical Laboratory Center, Beijing Youan Hospital, Capital Medical University, Beijing, China

**Keywords:** STIP1, hepatocellular carcinoma, AFP-negative hepatocellular carcinoma, diagnostic value

## Abstract

**Objective:**

This study aimed to investigate the diagnostic value of stress-induced phosphoprotein 1 (STIP1) in serum for hepatocellular carcinoma (HCC) and alpha-fetoprotein (AFP)–negative HCC (ANHC).

**Methods:**

In this study, serum samples were collected from 158 HCC patients and 63 non-HCC patients. Logistic regression analysis was performed to identify independent risk factors associated with HCC and ANHC. The diagnostic values of each index for HCC and ANHC were analyzed using receiver operating characteristic (ROC) curve analysis.

**Results:**

The STIP1, des-γ-carboxy prothrombin (DCP), and AFP levels were higher in the HCC groups than in the non-HCC groups (*P* < .05). Age, DCP, STIP1, and hepatitis B virus infection were independent predictors of HCC (*P* < .05). The diagnostic value of STIP1 for HCC was higher than that of DCP. Additionally, age, STIP1, and hepatitis B virus infection were independent predictors for ANHC patients. The ROC curve exhibited an area under the curve value of 0.919 for STIP1, with a diagnostic cutoff value of 68.5 U/mL. Moreover, 36 ANHC patients and 19 AFP-negative non-HCC patients were included to validate the diagnostic model. A total of 20 patients had STIP1 levels greater than 68.5 U/mL, resulting in diagnostic accuracy of 67.3%, sensitivity of 55.6%, and specificity of 89.5%.

**Conclusion:**

STIP1 demonstrates excellent diagnostic value for HCC and ANHC.

## Introduction

Hepatocellular carcinoma (HCC) is one of the most malignant tumors, with a high morbidity and high mortality.^1,2^ Every year, HCC causes the deaths of more than 700,000 individuals worldwide.^2^ Accounting for around 90% of primary liver cancers, HCC is the most common type and is especially prevalent in Asian countries, contributing to 75%-80% of cases globally.^3^ Despite a substantial decline in cancer mortality rates since 1991, the number of deaths related to HCC is still on the rise. The main factors contributing to the risk of developing HCC are liver cirrhosis and hepatitis B virus infection.^4^ Although surgical removal of the tumor and liver transplantation have shown efficacy in the management of HCC,^5^ the 5-year overall survival rate is still only 7%.^6^ The early detection of HCC remains a formidable challenge, invariably leading to poor patient outcomes. An accurate and timely diagnosis can enhance treatment effectiveness considerably and alleviate patient suffering.^7^ Currently, early diagnosis of HCC relies on the utilization of imaging techniques and serological examination.^8^ The National Comprehensive Cancer Network clinical practice guidelines suggest that high-risk HCC patients, specifically those with cirrhosis of any cause, should undergo abdominal ultrasound and alpha-fetoprotein (AFP) monitoring twice annually. Although ultrasound is the primary radiological screening technique, its effectiveness can be affected by the operator’s proficiency.^9^ It can be challenging to distinguish between malignant and benign nodules in cirrhotic liver, especially in cases where the nodules are small. The diagnostic accuracy of HCC has significantly improved due to the emergence of computed tomography (CT) and magnetic resonance imaging (MRI) methods. However, these techniques are costly and may not be suitable for mass screening.^10^

Alpha-fetoprotein was introduced in the 1960s as a serum marker for HCC. It remains the most commonly used biomarker for HCC worldwide.^11^ However, the sensitivity of AFP for diagnosing HCC is approximately 60% to 70%. Even with a low cutoff value (10-20 ng/mL), its specificity remains inadequate, resulting in many HCC patients being missed (30%-40%).^12^ In the case of AFP-negative HCC (ANHC), characterized by smaller tumor masses, imaging examinations are also prone to missing the diagnosis.^13^

ANHC accounts for approximately 30% to 40% of HCC. Its early diagnosis mainly relies on imaging tests such as ultrasound, CT, and MRI. However, ANHC mainly presents as a small HCC, often with mild clinical symptoms, lack of specificity, and inconspicuous imaging characteristics. This makes it more challenging to achieve early diagnosis and treatment in affected patients.^14^ ANHC currently lacks ideal biomarkers for early detection. However, the prognosis for patients in this group is generally favorable when treated promptly.^12^ Therefore, increasing emphasis on ANHC and actively exploring more effective diagnostic methods are crucial.

In recent years, significant progress has been made in identifying potential serum markers for ANHC diagnosis. Researchers have reported promising findings regarding the utility of des-γ-carboxy prothrombin (DCP), AFP-L3, Golgi protein 73 (GP73), and human cervical pro-oncogene 1 (HCCR-1), among others. DCP is an abnormal prothrombin molecule produced by an acquired defect in the posttranslational carboxylation of the prothrombin precursor in malignant cells. AFP-L3 is a specific form of AFP that binds to lectin *Lens culinaris* agglutinin.^12^ These markers hold great potential in improving the accuracy and early detection of ANHC. Further studies and exploration of novel serum markers are warranted to enhance our understanding and clinical management of this disease.^15^ Currently, the primary serum markers used in clinical practice for diagnosing and monitoring HCC are DCP and AFP-L3. DCP is an adjunctive marker to AFP and is elevated in certain HCC patients with low AFP levels or who test negative for AFP.^16^ A clinical combination test of DCP and AFP has shown the potential to enhance the diagnostic accuracy of ANHC.^17^ However, the use of DCP in clinical diagnosis also has its limitations, such as the risk of elevated DCP levels in benign liver diseases and other forms of cancer, which may necessitate additional tests combining multiple diagnostic indicators and imaging exams. Additionally, there are cases where DCP levels do not increase in specific individuals.^17^ Therefore, finding new tumor markers to diagnose ANHC is still necessary.

Phosphorylation stress-induced protein 1 (also known as stress-induced phosphoprotein 1 or STIP1) is emerging as a research breakthrough in diagnostic markers for HCC. STIP1, also called the tissue protein of heat shock protein 70/90, serves as a cochaperone, facilitating the transfer of client proteins from heat shock protein 70 to the heat shock protein 90 chaperone system.^18^ STIP1 is crucial in various essential processes besides its role as a tissue protein. It is actively involved in transcription regulation, cell cycle regulation, signal transduction, protein folding, and cell division.^19^ Previous studies have consistently demonstrated that elevated expression of STIP1 is commonly associated with an unfavorable prognosis in various malignant tumors, including HCC, pancreatic cancer, colorectal cancer, gastric cancer, and ovarian cancer.^20^ The diagnostic value of STIP1 in HCC and ANHC has not been reported. Our study aims to investigate the levels of STIP1 in the serum of HCC patients and explore its potential as a diagnostic marker in both HCC and ANHC.

## Methods

### Patients

The study collected serum samples from 158 HCC patients and 63 non-HCC patients (including 29 patients with chronic hepatitis, 19 healthy individuals, and 15 patients with liver cirrhosis) who were treated at Beijing Youan Hospital in August 2019. The diagnosis of HCC was based on the diagnostic criteria outlined in the Diagnosis and Treatment Guidelines for Primary Liver Cancer (2022 edition) issued by the National Health Commission of China. This study was approved by the Ethics Committee of Beijing Youan Hospital, and the utilization of residual serum samples from human subjects was exempted from informed consent (approval number: LL-2019-184-K). All procedures were conducted according to the 1964 Helsinki Declaration and its later amendments, or comparable ethical standards.

### Diagnostic Criteria for Primary Liver Cancer

- AFP ≥400 ng/mL in serum, which could exclude pregnancy, germ cell tumors, active liver disease, and metastatic liver cancer, and ultrasound or abdominal CT indicating a liver mass
- AFP <400 ng/mL in serum, and both ultrasound and abdominal CT indicating a liver mass, as well as ruling out other causes of elevated AFP, including pregnancy, active liver disease, germ cell tumors, and metastatic liver cancer
- Surgical and pathological confirmation of primary liver cancer

### Inclusion Criteria

- Age >18 years, with no limitations on sex, ethnicity, and region
- The diagnosis of HCC followed the Diagnosis and Treatment Guidelines for Primary Liver Cancer (2022 edition)
- Complete clinical medical records were available

### Exclusion Criteria

- Serum samples were deteriorated or less than 1 mL
- Patients who did not meet the diagnostic criteria
- Other diseases that could significantly affect the results of laboratory routine tests include severe infectious diseases, blood system diseases, immune-related diseases, and severe cardio-pulmonary-kidney diseases
- Incompleteness of medical records

### Collection of Clinical Data

Clinical data on patients, including sex, age, liver function (aspartate aminotransferase [AST], alanine aminotransferase [ALT], total bilirubin, serum albumin, γ-glutamyl transferase [GGT]) (using Siemens ADVIA2400), hepatitis virus markers (Roche cobas e801), serum tumor marker AFP (Roche cobas e601), DCP (Fujirebio Lumipulse G120), imaging examinations (CT, MRI), and pathological histology examinations, were collected from the hospital information management system of Beijing Youan Hospital, affiliated with Capital Medical University. The collected data were entered into an Excel spreadsheet. Additionally, 1 mL of fasting venous blood was collected from all patients, centrifuged at 1610*g* for 20 minutes to separate the serum, and stored at –80°C for future use.

### Detection of STIP1 in Serum

The STIP1 levels were measured using the human STIP1 ELISA kit (BIM) following these steps: Duplicate samples and standards were added to the micro ELISA strip plate. Protein standards were added appropriately, with the blank condition containing no sample. A total of 100 mL enzyme-labeled reagent was added to the standard and sample wells, followed by incubation at 37°C for 60 minutes. The MicroELISA plate was washed 4 times. A total of 50 μL coloring agent A and agent B were added to each well, gently mixed, and incubated at 37°C for 15 minutes. Then, 50 μL of stop solution was added to each well. The optical density at 450 nm was measured within 15 minutes using a Multiscan MK3 (Thermo Fisher Scientific).

### Statistical Methods

All statistical analyses were conducted using SPSS 21.0 software (IBM). Continuous variables were presented as mean plus or minus the standard error of the mean. The χ^2^ test, Fisher exact probability test, and Student *t*-test were used to determine significant differences between groups. The nonparametric Mann-Whitney *U* test was applied if the data were not homogeneous. Logistic regression analysis was performed to evaluate the significance of STIP1 in predicting HCC. The receiver operating characteristic (ROC) curve analysis was conducted to assess the value of serum STIP1 levels in predicting HCC. The Delong test was used to perform a significance test for the area under the curve (AUC). Data were considered statistically significant at *P* < .05.

## Results

### General Data Analysis

**Figure 1.**
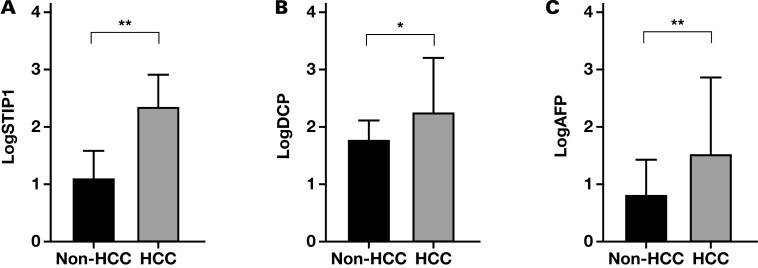
Comparison of HCC-related indicators between HCC and non-HCC groups. A, STIP1. B, DCP. C, AFP. AFP, alpha-fetoprotein; DCP, des-γ-carboxy prothrombin; HCC, hepatocellular carcinoma; STIP1, stress-induced phosphoprotein 1. *Significant difference at *P* < .05. **Significant difference at *P* < .001.

This study included 158 HCC and 63 non-HCC patients. In the HCC group, 76.6% were male, aged 27 to 85 years. Among them, 117 patients were infected with hepatitis B. In the non-HCC group, 69.8% were male, with an age range of 22 to 85 years, and 31 patients were infected with hepatitis B. There were no significant differences in age or sex between the 2 groups (*P* > .05). The general data of the patients, including ALT, AST, total bilirubin, albumin, GGT, AFP, DCP, STIP1, and whether they were infected with the hepatitis B virus, were subjected to statistical analysis. The results showed significant differences between the 2 groups regarding AFP, DCP, STIP1, and hepatitis B virus infection (*P* < .05), as shown in TABLE 1 and FIGURE 1.

**TABLE 1.** General Data Analysis of the Non-HCC and HCC Groups

Variable	Non-HCC (n = 63)	HCC (n = 158)	χ^2^/t/Z	*P*
Age, y, mean ± SD				
≤50	38.72 ± 1.46	42.10 ± 1.10	1.846	.071
>50	60.29 ± 1.28	62.99 ± 0.65	1.883	.065
Sex, n (%)			1.082	.192
Male	44 (69.8)	121 (76.6)		
Female	19 (30.2)	37 (23.4)		
ALT, IU/L	27 (18, 43)	24 (17, 37)	1.314	.189
AST, IU/L	28 (22, 56)	31 (23, 43)	0.077	.939
TBIL, μmol/L	19 (14.3, 29.3)	19.45 (12.78, 31.75)	0.203	.839
ALB, g/L	39.35 ± 1.087	38.53 ± 0.523	0.677	.5
GGT, U/L	45 (23, 104)	48 (30.75, 95. 5)	0.919	.358
HBsAg				
Negative, n (%)	32 (50.79)	41 (25.94)	12.568	.000^a^
Positive, n (%)	31 (49.21)	117 (74.05)		
AFP, ng/mL	3.94 (2.5, 10.17)	8.43 (3.41, 192.95)	3.819	.000^a^
DCP, mAU/mL	48 (34, 93)	74 (33, 573.5)	2.607	.009^b^
STIP1, U/mL	15.1 (8.6, 28.3)	405.85 (141.58, 554.9)	9.925	.000^a^

AFP, alpha-fetoprotein; ALB, albumin; ALT, alanine aminotransferase; AST, aspartate aminotransferase; DCP, des-γ-carboxy prothrombin; GGT, γ-glutamyl transferase; HBsAg, hepatitis B surface antigen; HCC, hepatocellular carcinoma; STIP1, stress-induced phosphoprotein 1; TBIL, total bilirubin.

^a^ *Significant difference at* P *< .001.*

^b^ *Significant difference at* P* < .05.*

### Univariate and Multivariate Logistic Regression Analysis Between HCC and Non-HCC Groups

The multivariate logistic regression analysis included the factors that showed statistical significance in the univariate analysis (*P* < .05). The results showed that age, DCP, STIP1, and hepatitis B virus infection were independent predictors of HCC, and the differences were statistically significant (*P* < .05), as shown in TABLE 2.

**TABLE 2.** Univariate and Multivariate Analysis Between the Non-HCC and HCC Groups

Variable	Univariate		Multivariate	
	Odds ratio (95% CI)	*P*	Odds ratio (95% CI)	*P*
Age	1.054 (1.027-1.082)	**.**000^a^	1.061 (1.014-1.110)	.01^b^
Sex	0.708 (0.369-1.359)	.299	—	—
ALT	0.989 (0.980-0.997)	.009^b^	0.971 (0.936-1.008)	.121
AST	0.998 (0.995-1.001)	.185	—	—
Log AST	0.572 (0.217-1.507)	.258	—	—
TBIL	0.992 (0.984-1.000)	.048^b^	1.002 (0.982-1.023)	.814
ALB	0.984 (0.944-1.026)	.447	—	—
GGT	0.999 (0.997-1.001)	.195	—	—
HBsAg	2.946 (1.603-5.414)	**.**001^a^	7.069 (1.841-27.140)	**.**004^b^
AFP	1.003 (1.000-1.007)	.079	—	—
Log AFP	2.061 (1.401-3.032)	**.**000^a^	0.783 (0.302-2.028)	.614
DCP	1.002 (1.000-1.004)	**.**024^b^	1.003 (1.001-1.006)	**.**016^b^
STIP1	1.038 (1.017-1.059)	**.**000^a^	1.037 (1.01-1.066)	**.**007^b^

AFP, alpha-fetoprotein; ALB, albumin; ALT, alanine aminotransferase; AST, aspartate aminotransferase; DCP, des-γ-carboxy prothrombin; GGT, γ-glutamyl transferase; HBsAg, hepatitis B surface antigen; STIP1, stress-induced phosphoprotein 1; TBIL, total bilirubin.

^a^ *Significant difference at* P* < .001.*

^b^ *Significant difference at* P* < .05.*

### Evaluation of the Diagnostic Value of STIP1 and DCP in HCC

**Figure 2.**
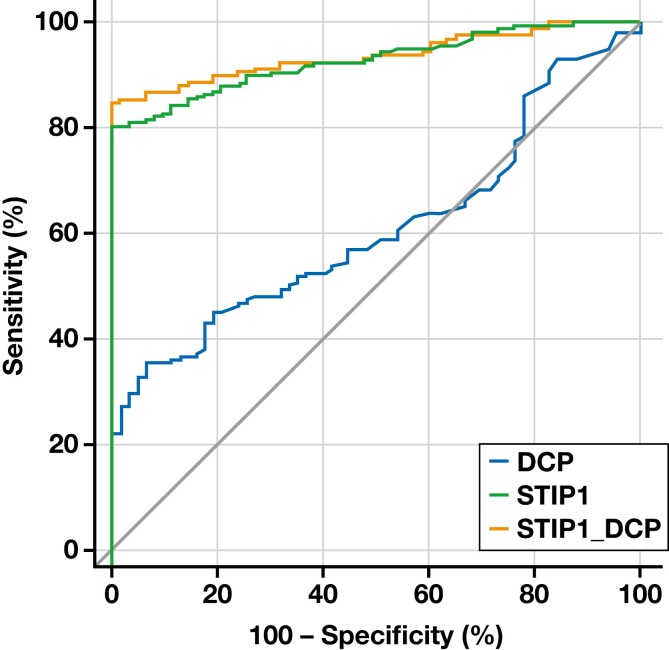
ROC curves of STIP1 and DCP for the diagnosis of HCC. DCP, des-γ-carboxy prothrombin; HCC, hepatocellular carcinoma; ROC, receiver operating characteristic; STIP1, stress-induced phosphoprotein 1.

The ROC curve was used to evaluate the diagnostic value of STIP1, DCP, and their combined indexes in HCC. The results showed that the diagnostic value of STIP1 in HCC was significantly higher than that of DCP (*P* < .05), and the diagnostic cutoff value was 78.85 U/mL. Notably, the combined index of STIP1+DCP had the largest area under the ROC curve, measuring 0.937, with a sensitivity of 84.8% and specificity of 98%, as shown in FIGURE 2 and TABLE 3. It is worth noting that the diagnostic value of STIP1 alone is not lower than that of multi-index combined detection, although the difference is not statistically significant (Z = 1.152, *P* = .249). Moreover, we divided the HCC group into 2 subgroups based on the cutoff value of STIP1 and showed that high levels of STIP1 may have a significant impact on AFP levels and nodule size, while the impact on tumor staging may not be significant, shown in Supplemental Table 1.

**TABLE 3.** ROC Curve Parameters for STIP1, DCP, and Combined Tests

Variance	Sensitivity (%)	Specificity (%)	Youden index	AUC	95% CI	*P*
DCP	35.44	93.65	0.2909	0.612	0.545-0.677	.0032^a^
STIP1	80.4	98	0.784	0.928	0.885-0.958	<.001^b^
STIP1+DCP	84.8	98	0.828	0.937	0.896-0.965	<.001^b^

AUC, area under the curve; DCP, des-γ-carboxy prothrombin; ROC, receiver operating characteristic; STIP1, stress-induced phosphoprotein 1.

^a^ *Significant difference at* P* < .05.*

^b^ *Significant difference at* P* < .001.*

### Univariate and Multivariate Analysis of Variance Between ANHC and Non-HCC Groups

Among the 73 patients with ANHC (<7 ng/mL), 83.56% were male, 83.6% were over 50 years old, and 23.3% were hepatitis B virus–infected. Univariate analysis revealed statistically significant differences between the 2 groups regarding age, ALT, AST, GGT, AFP, STIP1, and hepatitis B virus infection (*P* < .05). Further multivariate logistic analysis showed that age, STIP1, and hepatitis B virus infection were independent predictors of ANHC, as demonstrated in TABLE 4.

**TABLE 4.** Univariate and Multivariate Analysis Between Non-HCC Groups and ANHC

Variance	Non-HCC (n = 63)	ANHC (n = 73)	Univariate		Multivariate	
			Odds ratio (95% CI)	*P*	Odds ratio (95% CI)	*P*
Sex, male, %	69.84	83.56	0.456 (0.201-1.034)	.06	—	—
Age, y, n (%)						
≤50	25 (39.7)	12 (16.4)	3.344 (1.505-7.433)	**.**003^a^	1.079 (1.008-1.155)	**.**028^a^
>50	38 (60.32)	61 (83.6)				
ALT, IU/L	27 (18, 43)	22 (16, 32)	0.979 (0.963-0.995)	**.**013^a^	0.958 (0.875-1.048)	.349
AST, IU/L	28 (22, 56)	26 (19.5, 35)	0.978 (0.960-0.996)	**.**018^a^	1.001 (0.987-1.015)	.925
TBIL, μmol/L	19 (14.3, 29.3)	19.4 (11.8, 32.65)	0.992 (0.983-1.002)	.127	—	—
ALB, g/L	39.35 ± 1.09	38.6 ± 0.82	0.988 (0.945-1.032)	.575	—	—
GGT, U/L	45 (23, 104)	42 (28, 70)	0.995 (0.991-1.000)	**.**031^a^	1.005 (0.992-1.017)	.470
HBsAg, n (%)			3.4 (1.632-7.083)	**.**001^b^	13.790 (1.871-101.667)	**.**01^a^
Negative	32 (50.8)	56 (76.7)				
Positive	31 (49.2)	17 (23.3)				
AFP, ng/mL	3.94 (2.5, 10.17)	3.33 (2.36, 4.155)	0.837 (0.716-0.978)	.025^a^	0.848 (0.589-1.220)	.374
DCP, mAU/mL	48 (34, 93)	45 (29, 111)	1.001 (1.000-1.003)	.140	—	—
STIP1, U/mL	19.03 ± 1.96	364.58 ± 28.29	1.041 (1.015-1.067)	.002^a^	1.077 (1.023-1.133)	.005^a^

AFP, alpha-fetoprotein; ALB, albumin; ALT, alanine aminotransferase; ANHC, AFP-negative HCC; AST, aspartate aminotransferase; DCP, des-*γ*-carboxy prothrombin; GGT, γ-glutamyl transferase; HBsAg, hepatitis B surface antigen; HCC, hepatocellular carcinoma; STIP1, stress-induced phosphoprotein 1; TBIL, total bilirubin.

^a^ *Significant difference at* P* < .05.*

^b^ *Significant difference at* P* < .001.*

### Diagnostic Value of STIP1 in ANHC

**Figure 3.**
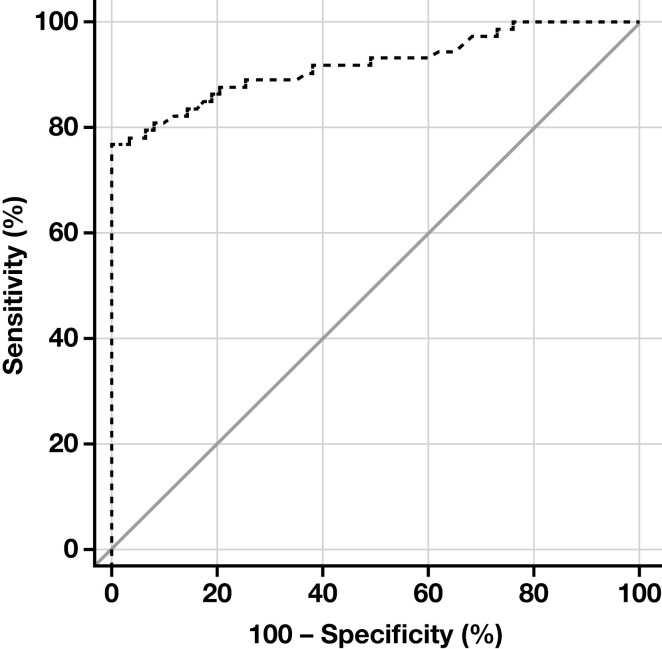
ROC curves of STIP 1 for diagnosis of ANHC. ANHC, alpha-fetoprotein–negative hepatocellular carcinoma; ROC, receiver operating characteristic; STIP1, stress-induced phosphoprotein 1. Sensitivity = 76.71%; specificity = 100%; area under the curve = 0.919.

In 73 ANHC patients, the diagnostic value of STIP1 for ANHC was evaluated using ROC curves. The findings demonstrated that STIP1 exhibited a strong diagnostic capability for ANHC (*P* < .001). The area under the ROC curve was 0.919, the diagnostic cutoff value was 68.5U/mL, and the sensitivity and specificity were 76.71% and 100%, respectively, as shown in FIGURE 3.

### Validation of the Diagnostic Model of STIP1 for ANHC

In addition, 36 cases of ANHC patients and 19 cases of ANHC non-HCC patients were selected for model validation. There were statistically significant differences in STIP1 levels and hepatitis B virus infection between the 2 groups, as shown in TABLE 5. Among the 36 patients, 20 patients had STIP1 levels greater than 68.5 U/mL, resulting in diagnostic accuracy of 67.3%, sensitivity of 55.6%, and specificity of 89.5%.

**TABLE 5.** Analysis of General Data for Non-HCC Groups and ANHC Groups

	Non-HCC (n = 19)	ANHC (n = 36)	χ^2^/t/Z	*P*
Age, y, mean ± SD	50.68 ± 3.117	57.08 ± 2.124	1.731	.089
Sex, n (%)				
Male	10 (52.6)	26 (72.2)	2.111	.233
Female	9 (47.4)	10 (27.8)		
HBsAg, n (%)				
Negative	18 (94.7)	12 (33.3)	18.912	.000^a^
Positive	1 (5.3)	24 (66.7)		
AFP	3.274 ± 0.377	3.205 ± 0.278	0.147	.884
STIP1	12.7 (9.1, 28.3)	252.6 (9.35, 604.75)	2.231	.026^b^

AFP, alpha-fetoprotein; ANHC, AFP-negative HCC; HBsAg, hepatitis B surface antigen; HCC, hepatocellular carcinoma; STIP1, stress-induced phosphoprotein 1.

^a^ *Significant difference at* P *< .001.*

^b^ *Significant difference at* P *< .05.*

## Discussion

HCC is one of the most common malignancies of the digestive system, with high morbidity and mortality. Its early diagnosis is crucial for timely treatment and improved survival rate. Although ultrasound, MRI, and other imaging techniques have greatly improved the accuracy of HCC diagnosis, its application is limited by its high cost, invasiveness, and insensitivity to small tumors. Therefore, the convenient, inexpensive, noninvasive, and reproducible detection of serum biomarkers plays an essential role in diagnosing HCC.^16^ AFP is a widely used biomarker for the diagnosis of HCC. However, its diagnostic accuracy is limited due to its high false-negative rate in detecting small and early-stage tumors. Zhang et al^21^ demonstrated that an AFP threshold of 400 ng/mL yielded a summary sensitivity of 0.32 (95% CI, 0.31-0.34) and specificity of 0.99 (95% CI, 0.98-0.99) by analyzing 29,828 articles from Medline and Embase databases. Marrero et al^22^ found that AFP >20 ng/mL had a sensitivity of 53% and a specificity of 90% for the early diagnosis of HCC. This means that even with a low cutoff value (eg, 10-20 ng/mL), there is a risk of misdiagnosing patients with ANHC.^23^ Furthermore, AFP may be elevated in some benign liver diseases, such as chronic hepatitis and cirrhosis without HCC. Currently, the use of AFP for early screening of HCC has been controversial.^24,25^ As a supplement to AFP, DCP has been used in clinical practice. It has been reported that a DCP level of ≥40 mAU/mL has a sensitivity of 64% and specificity of 89% in diagnosing early-stage liver cancer. The overall accuracy of this diagnostic approach is reported to be 86.3%.^26^ In the case of very early Barcelona Clinic Liver Cancer (BCLC 0) and early (BCLCA) HCC, the percentage of DCP (55.6% and 61.1%, respectively) was significantly higher than AFP (22.2% and 16.7%, respectively). In the middle and advanced stages of HCC (BCLCB/C), DCP also exhibited higher levels than AFP, although the difference was relatively minor.^16^

In our study, AFP and DCP were tested in 158 HCC patients. The results showed that the levels of AFP and DCP were significantly higher in the HCC group than the non-HCC group. Univariate analysis confirmed a strong correlation between log AFP and DCP with HCC. Furthermore, multivariate analysis revealed that DCP was an independent predictive factor for HCC. Results from the ROC curve analysis demonstrated that DCP had a specificity of 93.65% and a sensitivity of 35.44%.Our study also confirmed that AFP >400 ng/mL is consistent with the diagnosis of pathological examination in HCC (κ = 0.042, *P* = .013), with a specificity of approximately 100% and a sensitivity of 20.3% (Supplemental Table 2). In addition, we found that the specificity for diagnosing HCC with tumor nodules <2 cm when AFP > 400 ng/mL was 100%, whereas the sensitivity was 4%, due to a small sample size in the control group (Supplemental Table 3). These results emphasize the limitations of using AFP alone to diagnose HCC, particularly given a certain missed rate of ANHC.^27^ Patients with ANHC have a better prognosis than AFP-positive HCC patients. Studies have found that the 1-year, 3-year, and 5-year recurrence-free survival rates for the ANHC group were 78.1%, 57.5%, and 40.6%, respectively, whereas those for the AFP-positive group were 61.8%, 37.7%, and 31.4%, respectively. Correspondingly, the overall survival rates for the ANHC group were 94.4%, 83.8%, and 62.3%, respectively, and those for the AFP-positive group were 87.2%, 60.0%, and 36.7%, respectively.^25^ Therefore, diagnosing ANHC is essential to improving prognosis for HCC patients. Currently, the detection methods for ANHC are divided into serum biomarkers and imaging examinations. China’s Diagnosis and Treatment Guidelines for Primary Liver Cancer (2022), issued by the National Health Commission, recommends combining AFP-L3 and DCP to improve the diagnosis rate of early HCC. In our study, we screened a total of 73 ANHC patients, and no difference was found in DCP levels between the ANHC and non-HCC groups, which may be due to the limited sample size in the experiment.

Scholars have been actively dedicated to identifying ANHC biomarkers with significant diagnostic value. Shu et al^28^ used serum proteomics technology to discover 5 HCC biomarkers. Among these biomarkers, the combination of haptoglobin demonstrates significant diagnostic value for ANHC, with an impressive AUC of 0.763. Zhu et al^29^ revealed that serum heparin-binding cytokines exhibit an 80% positivity rate in ANHC. Furthermore, there is also some diagnostic value in ANHC for inhibitors of the Wnt pathway, such as Dickkopf1,^30^ adenylyl cyclase-associated protein 2,^31^ and latent transforming growth factor-beta-binding protein 1.^32^ However, more extensive research is required to confirm the diagnostic potential of these biomarkers in ANHC.

STIP1 is a multifunctional protein that plays a crucial role in various cellular processes. Encoded by the STIP1 gene, it is widely expressed in different tissues and cells. STIP1 has been identified as a key player in cancer development and metastasis. Studies suggest that STIP1 regulates tumor cell growth and invasiveness, contributing to tumor progression.^33^ Furthermore, STIP1 is also implicated in cancer-related factors such as drug resistance and poor prognosis. Ma et al^18^ conducted studies and found that STIP1 levels were significantly elevated in the HCC group, suggesting its potential as an early diagnostic marker for HCC. Chen et al^33^ reported that serum STIP1 level was positively correlated with the malignancy degree and tumor size in HCC, indicating STIP1 as a potential biomarker for HCC diagnosis and prognostic evaluation.

Our study revealed that the STIP1 level was significantly higher in the HCC group than in the non-HCC group. Univariate analysis demonstrated that STIP1 and tumor markers log AFP and DCP were associated factors of HCC, whereas multivariate logistic analysis showed STIP1 and DCP as independent predictors of HCC. Further results from the ROC curve analysis demonstrated that STIP1 combined with DCP exhibited a sensitivity of 84.8% and specificity of 100%. Interestingly, this experiment revealed that the diagnostic value of STIP1 alone was comparable to that of multi-index combination detection, although without statistical significance (Z = 1.152, *P* = .249). In conclusion, STIP1 holds significant diagnostic value in HCC. We classified 158 HCC patients based on different levels of STIP1 and observed that a majority of those with high STIP1 levels were in the subgroup with AFP <400 ng/mL and nodules >2 cm, indicating that STIP1 might hold greater diagnostic value for HCC patients with low AFP levels. Previous research shows that larger nodules may indicate a higher degree of tumor invasiveness, thereby suggesting a potential role for STIP1 in the progression of HCC. For the first time, our study confirmed the significant diagnostic value of STIP1 in ANHC. We screened 73 ANHC patients and found that age, ALT, AST, GGT, AFP, STIP1, and hepatitis B virus infection were significantly associated with ANHC. At the same time, DCP did not show such an association, possibly due to the small sample size. Multivariate analysis demonstrated that STIP1 was also an independent predictor of ANHC. The ROC curve results indicated that STIP1 exhibited excellent diagnostic value for ANHC (*P* < .0001). The area under the ROC curve was 0.919, with a diagnostic cutoff value of 68.5 U/mL, sensitivity of 76.71%, and specificity of 100%. Additionally, the validation of the ANHC diagnostic model yielded a diagnostic accuracy of 67.3%, sensitivity of 55.6%, and specificity of 89.5%.

In conclusion, STIP1 shows significant potential as a diagnostic biomarker for HCC and ANHC, providing a new theoretical foundation for diagnosing such diseases. However, further validation and expansion of the sample size are necessary to confirm the diagnostic value of STIP1 and establish a new laboratory basis for the early clinical diagnosis of HCC.

## Supplementary Material

lmae033_suppl_Supplementary_Tables

## Abbreviations

STIP1: stress-induced phosphoprotein 1
HCC: hepatocellular carcinoma
AFP: alpha-fetoprotein
ANHC: AFP-negative HCC
ROC: receiver operating characteristic
DCP: des-γ-carboxy prothrombin
CT: computed tomography
MRI: magnetic resonance imaging
GP73: Golgi protein 73
HCCR-1: human cervical pro-oncogene 1
AUC: area under the curve
ALT: alanine aminotransferase
AST: aspartate aminotransferase
TBIL: total bilirubin
ALB: albumin
GGT: γ-glutamyl transferase
BCLC: Barcelona Clinic Liver Cancer
