# Supplementary material for: Diagnostic value of serum STIP1 in HCC and AFP-negative HCC
Source: Lab Med. 2024 May 23;55(6):700–7. doi: 10.1093/labmed/lmae033 (PMC11532616; doi:10.1093/labmed/lmae033)
Supplement: lmae033_suppl_Supplementary_Tables [file lmae033_suppl_supplementary_tables.doc]

**Table S1** A comparison of clinical data from 158 HCC patients

|  | STIP1  ≤78.85 U/mL | STIP1  >78.85 U/mL | χ２ | P 值 |
| --- | --- | --- | --- | --- |
| AFP, ng/mL |  |  |  |  |
| <400 | 31 | 95 |  | 0.001* |
| ≥400 | 0 | 32 |  |  |
| Tumor size,cm |  |  |  |  |
| ≤2 | 15 | 35 | 4.997 | 0.032* |
| >2 | 16 | 92 |  |  |
| Stage |  |  |  |  |
| I-II | 23 | 72 | 3.183 | 0.101 |
| III-IV | 8 | 55 |  |  |

*indicates a significant difference at *P*<0.05.

**Table S2**  The comparison of AFP and pathological methods in diagnosing HCC

|  | Pathology | |  |
| --- | --- | --- | --- |
|  | + | - |  |
| AFP>400ng/mL | 32 | 0 | 32 |
| AFP<400ng/mL | 126 | 15 | 141 |
| Total |  |  | 173 |

**Table S3**  The comparison of AFP and pathological methods in diagnosing small HCC

|  | Pathology | |  |
| --- | --- | --- | --- |
|  | + | - |  |
| AFP>400ng/mL | 2 | 0 | 2 |
| AFP<400ng/mL | 48 | 15 | 63 |
| Total |  |  | 65 |
